# Divided Attention and Processes Underlying Sense of Agency

**DOI:** 10.3389/fpsyg.2016.00035

**Published:** 2016-01-28

**Authors:** Wen Wen, Atsushi Yamashita, Hajime Asama

**Affiliations:** Department of Precision Engineering, The University of TokyoTokyo, Japan

**Keywords:** attention, cognitive load, comparator model, dual task, performance, sense of agency, working memory

## Abstract

Sense of agency refers to the subjective feeling of controlling events through one’s behavior or will. Sense of agency results from matching predictions of one’s own actions with actual feedback regarding the action. Furthermore, when an action involves a cued goal, performance-based inference contributes to sense of agency. That is, if people achieve their goal, they would believe themselves to be in control. Previous studies have shown that both action-effect comparison and performance-based inference contribute to sense of agency; however, the dominance of one process over the other may shift based on task conditions such as the presence or absence of specific goals. In this study, we examined the influence of divided attention on these two processes underlying sense of agency in two conditions. In the experimental task, participants continuously controlled a moving dot for 10 s while maintaining a string of three or seven digits in working memory. We found that when there was no cued goal (no-cued-goal condition), sense of agency was impaired by high cognitive load. Contrastingly, when participants controlled the dot based on a cued goal (cued-goal-directed condition), their sense of agency was lower than in the no-cued-goal condition and was not affected by cognitive load. The results suggest that the action-effect comparison process underlying sense of agency requires attention. On the other hand, the weaker influence of divided attention in the cued-goal-directed condition could be attributed to the dominance of performance-based inference, which is probably automatic.

## Introduction

The ubiquitous experience of a subjective feeling of control over the outcome of events through one’s behavior refers to sense of agency. When one uses external tools, such as a keyboard to type a text message, a sense of agency in producing letters on the screen may have resulted because changes on the screen precisely match the individual’s predictions (Frith et al., 2000; Blakemore et al., 2002). Such matching processes linked to sense of agency can be explained through the comparator model, according to which, a predicted state is generated from an efference copy of one’s motor command and is compared with actual sensory information; a match results in sense of agency while a mismatch diminishes sense of agency (Blakemore et al., 1998, 1999; Frith et al., 2000; Wolpert and Flanagan, 2001). In the present study, this comparison process is termed the *action-effect comparison*. For example, sense of agency increases considerably when there is a match between actual sensory feedback and predictions based on prior experience (Sato and Yasuda, 2005). Further, the comparator model was also able to show that the abnormal sense of agency experienced by schizophrenic patients is linked to difficulty with generating predictions (Cahill et al., 1996; Frith et al., 2000).

In addition to the feed-forward processes emphasized in comparator models, recent studies have highlighted the role of reconstructive processes (Wegner, 2003; Wegner et al., 2004; Metcalfe and Greene, 2007; Synofzik et al., 2008; Wen et al., 2015c). Wegner’s reconstructive account of the sense of agency suggests that when a thought appears in consciousness before an action is executed (the priority principle), is consistent with the action (the consistency principle), and there are no alternative causes of the action (the exclusivity principle), the individual believes that he/she is the agent of that action (Wegner, 2003). Although Wegner’s theory was intended to provide a reconstructive account of conscious will or action (i.e., conscious will is a result of the principles outlined by Wegner), it also influences the theory on sense of agency for external events. That is, sense of agency is not only generated from predictive processes—predicting that an effect will occur—but also from reconstructive processes, such as making inferences after an effect occurs. Recent studies have reported that reconstructive processes significantly influence sense of agency (Wegner et al., 2004; Metcalfe and Greene, 2007; Metcalfe et al., 2013; Wen et al., 2015c). For example, inference based on performance is a reconstructive process, and a previous study found that individuals felt a stronger sense of agency when they performed well even when their actual control was weakened (Wen et al., 2015c).

Thus, both action-effect comparison processes and performance-based inference are considered to contribute to the sense of agency, and the strength of these underlying processes may differ in different conditions (Moore and Fletcher, 2012; Synofzik et al., 2013; Wen et al., 2015c). Nonetheless, it is still unclear whether these processes are automatic or controlled by attentional demand. Attention is the process of focusing one’s consciousness on specific stimuli or processes; however, attentional resources are considered to be limited (Baddeley, 2003). If people have to perform two or more attention-demanding tasks simultaneously, their attention is divided between tasks, potentially resulting in inhibitory effects in all tasks. Research suggests that there are two types of processes based on attentional demand (Schneider and Shiffrin, 1977; Schneider and Chein, 2003); controlled processes are temporary sequences of memory nodes activated under the control of attention, while automatic processes involve the automatic activation of a sequence of memory nodes without attentional control (Schneider and Shiffrin, 1977). Typically, automatic processes are fast, parallel, robust, and less controllable, with low attentional demands, while controlled processes are slow, serial, effortful, and brittle (Schneider and Chein, 2003). Neuroscience research has provided evidence for a dissociation between controlled and automatic processes (Hahne and Friederici, 1999; Schneider and Chein, 2003). In addition, if a task involves both controlled and automatic processes, insufficient attentional resources may result in changes in the dominance of underlying processes; typically, controlled processes would be impaired while automatic processes would dominate (e.g., Gardiner and Parkin, 1990; Yonelinas, 2002; Evans, 2008).

Previous studies on the development of sense of agency suggest that both implicit and explicit cues can influence the judgment of agency (Aarts et al., 2005; Linser and Goschke, 2007; Wenke et al., 2010). Explicit cues refer to stimuli that can be consciously perceived, while implicit cues refer to stimuli that cannot be consciously perceived (e.g., visual stimuli presented for 10–20 ms). A two-step theory of agency distinguishes between judgments and feelings of agency, suggesting that the former is an explicit, conceptual, interpretative judgment of being the agent, whereas the latter is non-conceptual, low-level, and implicit understanding (Synofzik et al., 2008). However, it is unclear whether the judgment or feeling of agency is controlled or automatic processes. Processes that are influenced by implicit cues may also require attentional resources, because implicit cues may influence the perception of action effects (e.g., they may be easier to perceive or are perceived more vividly; Bar and Biederman, 1998; Stenner et al., 2014), and therefore affect sense of agency. However, the comparison of action and effect itself may require attention. On the other hand, processes that use explicit cues—such as performance or contextual cues—could be performed automatically without cognitive resources (i.e., do not rely on attentional resources; De Neys, 2006). In addition, the theory posits that implicit and explicit processes do not correspond to automatic and controlled processes, because the latter classification is based on attention, not consciousness. For example, although controlled processes are consciously performed, conscious processes may be controlled or automatic.

Further, a prior study has reported that sense of agency decreased with higher cognitive load when the action and effect were consistent and the temporal interval between them was no longer than 100 ms (Hon et al., 2013). However, in Haggard et al.’s (2002) study on the intentional binding effect—which refers to the temporal compression of the interval between one’s voluntary action and the corresponding effect, considered to reflect implicit aspects of sense of agency—binding effects were stronger when participants were unable to focus their attention on a specific event (Haggard and Cole, 2007), indicating that divided attention might promote implicit sense of agency. To the best of our knowledge, no study has investigated the attentional demands of both perceptual and higher-order cognitive processes underlying sense of agency.

In the present study, we attempted to examine the influence of divided attention on both the action-effect comparison process and performance-based inference that underlie sense of agency, in order to determine whether these processes are controlled or automatic. We employed a dual-task paradigm to ensure participants’ divided attention for the agency task. Participants continuously controlled a target on a computer screen for a specific duration, and then rated their sense of agency on the target’s location while doing a memory task that simultaneously required continuous attention. The dual-task paradigm has been widely used to examine whether or not a process has attentional demands (Baddeley et al., 1984; Fernandes and Moscovitch, 2000, 2002; Schneider and Chein, 2003; Pouliot and Gagnon, 2005). Moreover, participants controlled the target with or without a cued goal in two conditions. In the no-cued-goal condition, participants controlled a target and moved it on the screen according to their own will. Because a specific goal was not present in the no-cued-goal condition, performance feedback was not provided to participants. Although actions may be driven by internal goals, active performance-based inference would be much lower in this condition than when a cued-goal and feedback of attainment of the goal were present wherein the judgment of agency would primarily rely on how similar the change in the direction of the target’s movement was to participants’ predictions of the movement of the dot. Contrastingly, in the cued-goal-directed condition, participants were instructed to move the target to a specific destination as much as possible. Since feedback of goal attainment was provided to participants, performance-based inference would play an important role in the judgment of agency (Wen et al., 2015b,c). If the action-effect comparison process involves attentional control, sense of agency would be impaired by high cognitive load, and the decrease in sense of agency in the no-cued-goal condition would be larger. If the comparison process is automatic, but inference requires attention, sense of agency in the cued-goal-directed condition would be more strongly influenced by divided attention than in the no-cued-goal condition, because performance-based inference is more likely to have a greater influence on sense of agency in the cued-goal-directed condition than in the no-cued-goal condition. If both comparison and inference processes require attention, sense of agency in no-cued-goal and cued-goal-directed conditions would be impaired by high cognitive load. However, in this case, a separate condition with no visible target and only feedback on goal achievement is needed to further examine whether performance-based inference is automatic or controlled. Moreover, the interval between actions and effects was used to vary the difficulty of the experimental task, as delays would not only increase the task difficulty (i.e., directing the moving dot into the destination), but would also make comparisons between actions and their corresponding effects more difficult.

## Materials and Methods

### Participants

A total of 18 students with normal or corrected-to-normal visual acuity participated in the experiment and received monetary compensation for participation. Participants’ mean age was 25.7 years (*SD* = 3.6, range: 22–33). All participants were right-handed. The experiment was approved by the ethics committee of the Faculty of Engineering at the University of Tokyo and written informed consent was obtained from all participants.

### Task and Procedure

**FIGURE 1:**
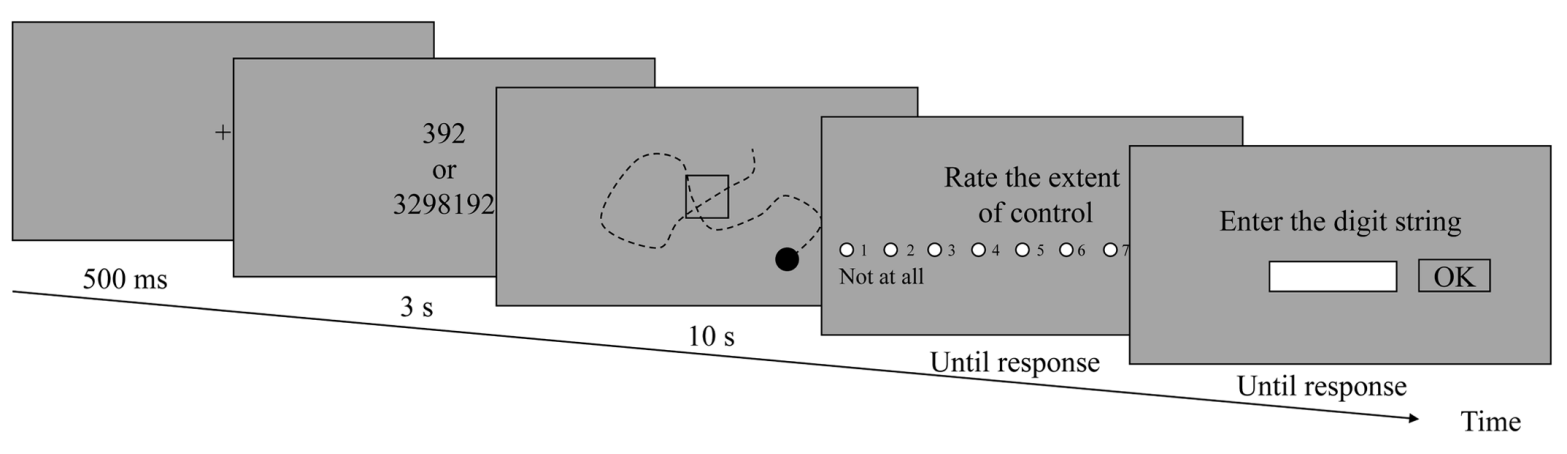
**Timeline of an experimental task trial in the cued-goal-directed condition.** Participants were instructed to maintain a 3- or 7-digit string in their mind while directing the moving dot into the square as quickly as possible by pressing the left and right keys to change the direction in which the dot traveled. Ten seconds after the appearance of the dot, participants used a mouse to rate the extent to which they felt that the dot was under their control, and then inputted the memorized digital string with the keyboard. In the no-cued-goal condition, the square was not presented, and participants were instructed to control the dot according to their will.

In each trial of the experimental task (**Figure 1**), participants were first shown a black cross at the center of a 597 mm × 336 mm (width × height) screen with a gray background for 500 ms. Then, a string of three (low-load condition) or seven (high-load condition) randomly generated digits replaced the cross and was presented for 3 s. Participants were required to maintain the digit string in their mind during the trial. After the digit string disappeared, a 5-mm black dot appeared at a random position on the screen for 10 s and moved at a speed of 124 mm/s. The original direction of the dot was randomly generated and differed between trials. The direction of the dot did not change until participants pressed the left or right key during the trial. The dot bounced back when it reached the screen borders. Participants were instructed to repeatedly press the left or right key on a keyboard with their index and middle fingers of their right hand to change the direction of the moving dot. The direction of the dot turned 20° clockwise with a right key press and 20° counterclockwise with a left key press.

In the no-cued-goal condition, only a moving dot appeared on the screen. Participants were asked to pressing the left and right keys continuously, according to their will in order to determine whether the direction of the moving dot responded to their key presses. They were requested to avoid pressing only one key. They were instructed that they could move the dot to any position of their choosing on the screen. In the cued-goal-directed condition, a 30-mm empty square with a black border appeared at the center of the screen together with the moving dot. Participants were instructed to determine their control over the moving dot. Furthermore, they were instructed to direct the dot into the square as quickly as possible. When the dot had crossed the border to enter the square, the color of the dot turned red to indicate its arrival, and the dot continued moving. After leaving the square, the color of the dot turned black. Participants were instructed to direct the dot back into the square (if the dot went out) as often as possible within the given time (10 s). After the task, participants rated the extent to which they felt that the dot was under their control on a 9-point scale (1 = not at all; 9 = a lot) by clicking on-screen radio buttons using a mouse; they then had to input the digit string that was presented at the beginning of the trial using the keyboard. There were three possible delays (100, 400, and 700 ms) between participants’ key presses and the dot’s response. The delay was consistent within each trial and varied randomly between trials. Participants were told that both the memory test and the dot-controlling task were important and that they should divide their attention equally between the two tasks. Participants were not given feedback on their accuracy in the memory task.

Ratings on agency, number of arrivals at the destination (in the cued-goal-directed condition), and responses in the memory task were recorded for each trial. The number of arrivals at the destination reflects participants’ task performance in the cued-goal-directed condition. Accuracy of responses in the memory task reflects the engaged cognitive resources.

Participants were tested individually in a quiet room; they were seated on a chair positioned about 50 cm from a 27-in LCD monitor with a resolution of 1,920 pixels × 1,080 pixels. After receiving instructions, participants were administered 10 practice trials with random delays and conditions. Each participant then completed 120 experimental trials, comprising 10 trials for each delay condition (100, 400, and 700 ms), each load condition (low- and high-load condition), and each goal condition (no-cued-goal and cued-goal-directed condition), presented in a random order. The 120 trials were divided into two blocks, each containing 60 trials. Participants took 5-min breaks between blocks. On average, the experiment lasted for 60 min.

## Results

### Agency Rating

**FIGURE 2:**
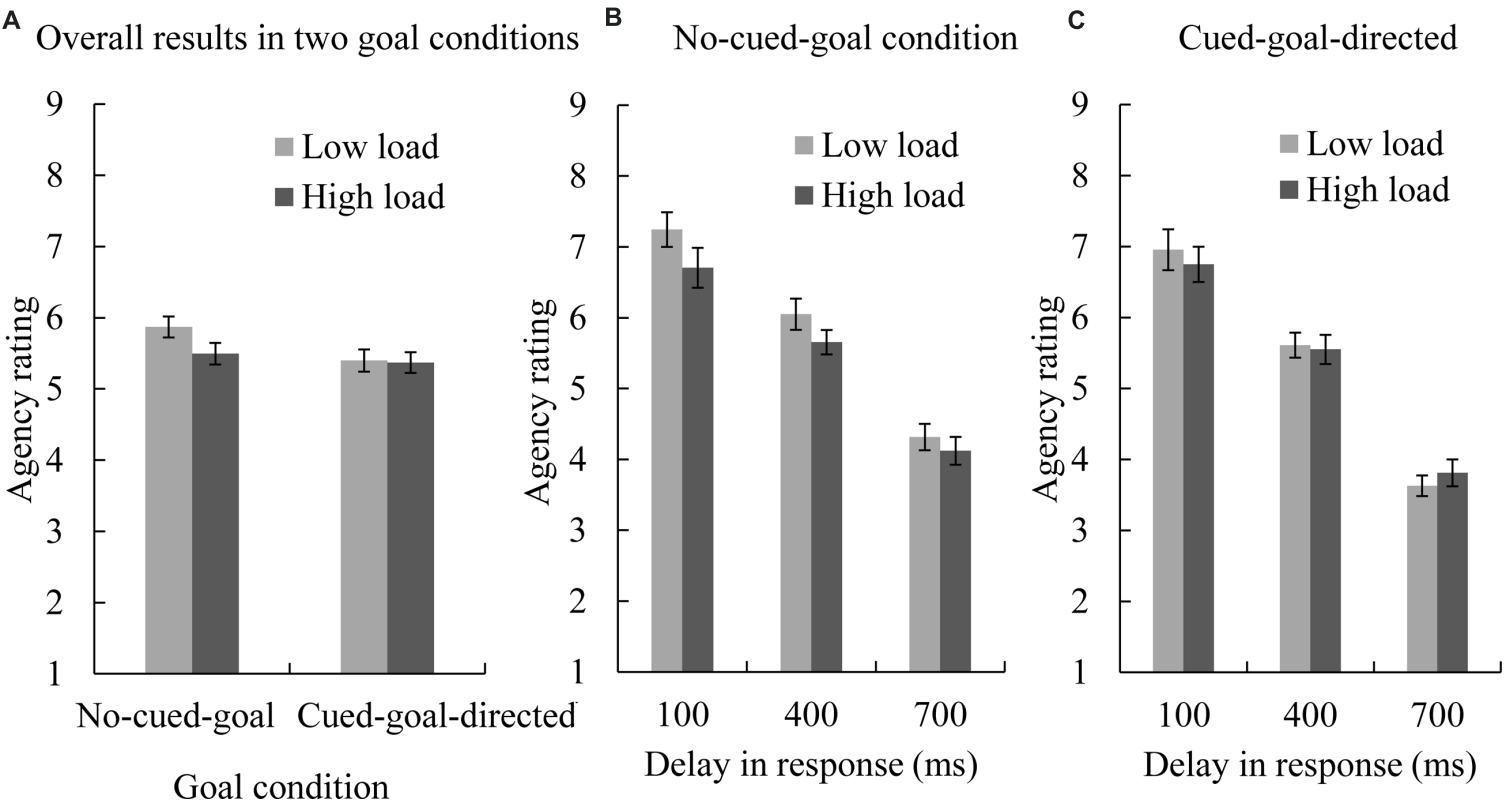
**Mean agency ratings in each condition.** Error bars represent standard errors of the mean. **(A)** Shows mean agency ratings in the no-cued-goal and cued-goal-directed conditions; **(B)** shows mean agency ratings in the no-cued-goal condition by delay and cognitive load; **(C)** shows mean agency ratings in the cued-goal-directed condition by delay and cognitive load; agency ratings were impaired by high cognitive load in the no-cued-goal condition, but were not influenced by cognitive load in the cued-goal-directed condition.

A 2 (no-cued-goal or cued-goal-directed condition) × 2 (low- or high-load condition) × 3 (100, 400, or 700 ms delay) repeated-measures ANOVA on the agency rating scores was conducted to examine the overall experimental design. The main effect of goal was significant [*F*(1,17) = 11.05, *p* = 0.004, $\eta_{p}^{2}$ = 0.39]; participants gave lower agency ratings in the cued-goal-directed condition compared to in the no-cued-goal condition (cued-goal-directed: *M* = 5.38; no-cued-goal: *M* = 5.68). The main effects of cognitive load and delay were also significant [*F*(1,17) = 11.46, *p* = 0.004, $\eta_{p}^{2}$ = 0.40; *F*(2,34) = 85.75, *p* = 0.000, $\eta_{p}^{2}$ = 0.84, respectively]. Participants gave lower agency ratings in the high cognitive load condition compared to the low cognitive load condition (high load: *M* = 5.43; low load: *M* = 5.63). *Post hoc* comparisons (Bonferroni-corrected) on delay showed that participants provided lower agency ratings in the longer delay conditions compared to shorter delays (100-ms delay: *M* = 6.91; 400-ms delay: *M* = 5.72; 700-ms delay: *M* = 3.97; for all differences between delay conditions, *p*s = 0.000). Furthermore, the only significant interaction was between goal and cognitive load [*F*(1,17) = 9.07, *p* = 0.008, $\eta_{p}^{2}$ = 0.35, **Figure 2A**]. In order to clarify this interaction and the influence of cognitive load in trials with and without a goal, we re-examined our results for the no-cued-goal and cued-goal-directed conditions separately.

In the no-cued-goal condition, the main effects of load and delay were both significant [*F*(1,17) = 49.29, *p* = 0.000, $\eta_{p}^{2}$ = 0.74; *F*(2,34) = 59.46, *p* = 0.000, $\eta_{p}^{2}$ = 0.78, respectively; **Figure 2B**], while the interaction between load and delay was not significant [*F*(2,34) = 1.11, *p* = 0.088, $\eta_{p}^{2}$ = 0.06]. Participants gave lower ratings in the high cognitive load condition compared to the low cognitive load condition (high load: *M* = 5.49; low load: *M* = 5.87). *Post hoc* comparisons (Bonferroni-corrected) for delay showed that participants felt that they had less control in the longer delay conditions relative to shorter delay conditions (100-ms delay: *M* = 6.98; 400-ms delay: *M* = 5.85; 700-ms delay: *M* = 4.22; differences between all delay conditions were significant, *p*s = 0.000).

In contrast, in the cued-goal-directed condition, only the main effect of delay was significant [*F*(2,34) = 86.26, *p* = 0.000, $\eta_{p}^{2}$ = 0.84; **Figure 2C**). *Post hoc* comparisons (Bonferroni-corrected) for delay showed that participants felt that they had less control in the longer delay conditions relative to shorter delay conditions (for the 100-ms delay condition, *M* = 6.85; for the 400-ms delay condition, *M* = 5.58; for the 700-ms condition, *M* = 3.72; differences between all delay conditions were significant, *p*s = 0.000). The availability of cognitive resources had no significant effect on agency ratings and did not interact with delay [main effect of load: *F*(1,17) = 0.07, *p* = 0.794, $\eta_{p}^{2}$ = 0.00; interaction between load and delay: *F*(2,34) = 1.62, *p* = 0.213, $\eta_{p}^{2}$ = 0.09]. Therefore, when goals were present during the dot-controlling task, higher cognitive load did not affect agency ratings.

### Performance of Main and Secondary Tasks

**FIGURE 3:**
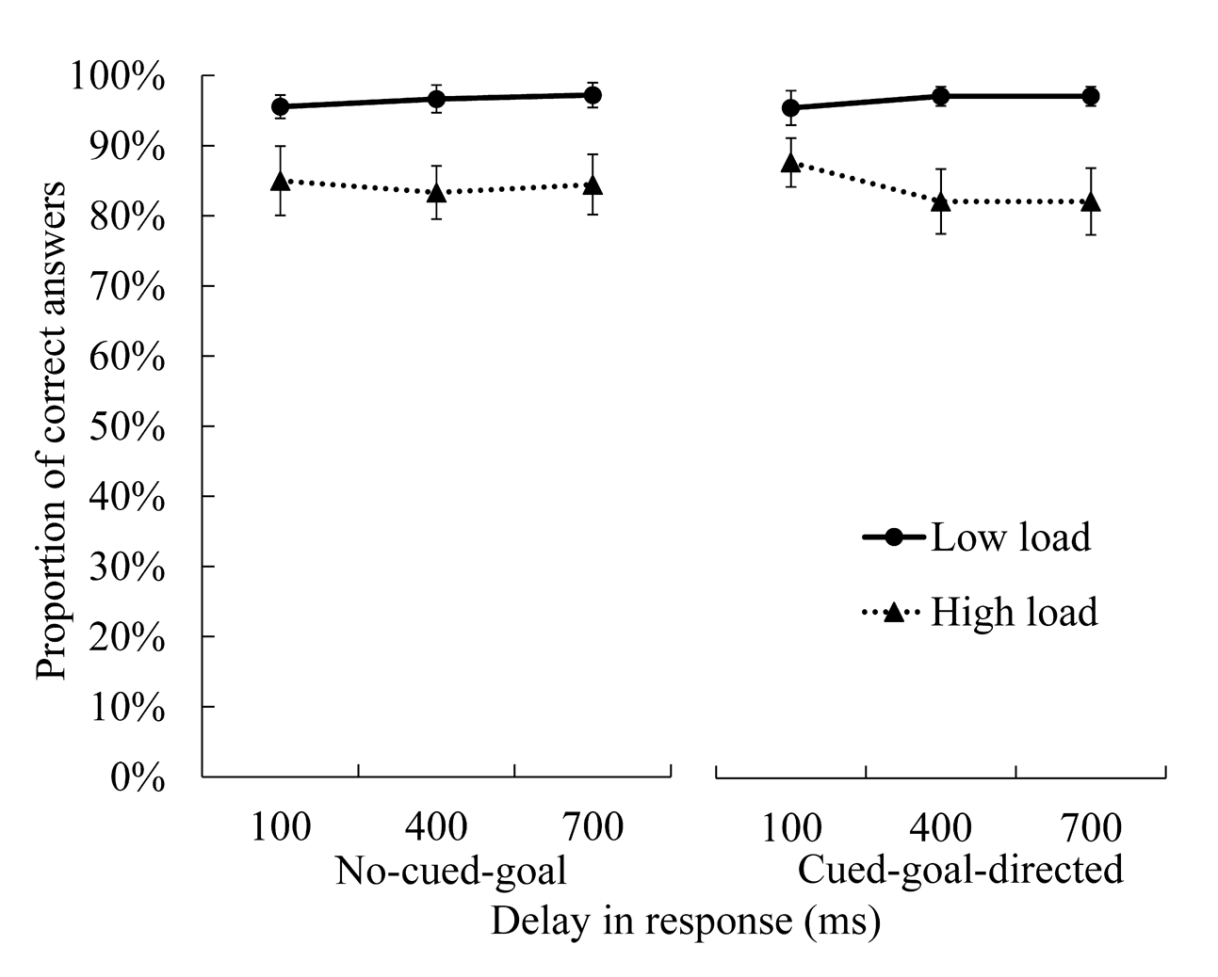
**Proportion of correct responses in the digit memory task.** Error bars represent standard errors of the mean. Performance in the memory task was poorer in the high cognitive load condition than that in the low cognitive load condition.

**FIGURE 4:**
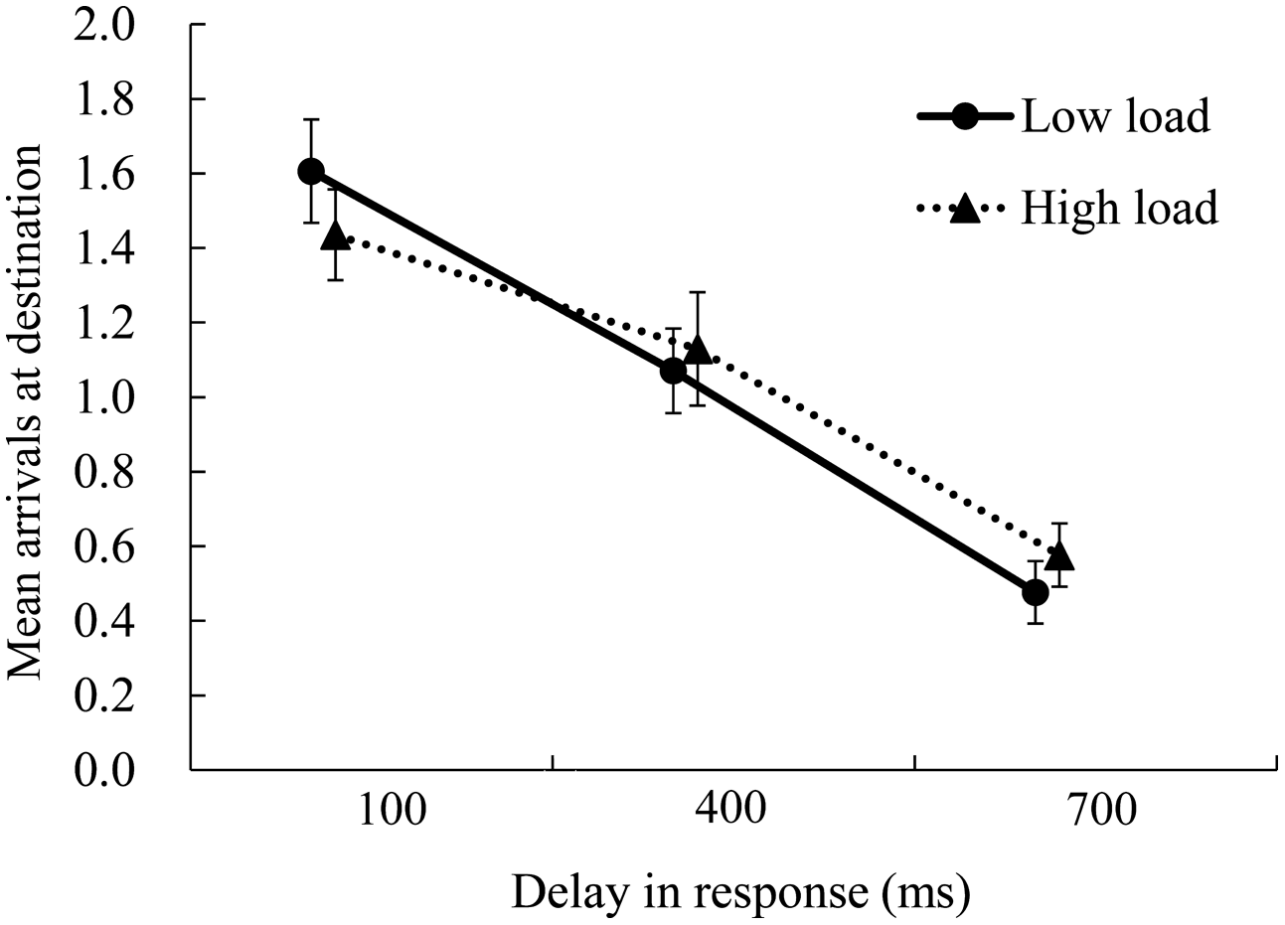
**The average number of arrivals at the destination per trial in the experimental task.** Error bars represent standard errors of the mean. Performance was impaired by delays in the dot’s response, but was not influenced by cognitive load.

The proportion of correct answers for the digit memory task and the average number of arrivals in each trial in the cued-goal-directed condition are shown in **Figures 3** and **4**, as indices of performance in the memory task (i.e., secondary task) and experimental (i.e., main task), respectively. Regarding memory of digit strings, the main effect of load was significant [*F*(1,17) = 13.76, *p* = 0.002, $\eta_{p}^{2}$ = 0.45], but the main effects of goal and delay, and the interactions between all three factors were not significant [main effect of goal: *F*(1,17) = 0.00, *p* = 1.000, $\eta_{p}^{2}$ = 0.05; main effect of delay: *F*(2,34) = 0.448, *p* = 0.643, $\eta_{p}^{2}$ = 0.03; interaction between goal and load: *F*(1, 17) = 0.02, *p* = .892, $\eta_{p}^{2}$ = 0.00; interaction between goal and delay: *F*(2,34) = 0.27, *p* = 0.765, $\eta_{p}^{2}$ = 0.02; interaction between load and delay: *F*(2,34) = 2.71, *p* = 0.081, $\eta_{p}^{2}$ = 0.14; interaction between goal, load, and delay: *F*(2,34) = 0.36, *p* = 0.703, $\eta_{p}^{2}$ = 0.02]. Participants’ accuracy in the memory task was better in the low-load condition than in the high-load condition (low-load condition: proportion of correct answer = 97%; high-load condition: proportion of correct answer = 84%). It was more difficult to memorize seven digits while controlling the moving dot than to memorize three digits; the former may have required more cognitive resources. Regarding performance of the experimental task, the main effect of delay was significant [*F*(2,34) = 79.81, *p* = 0.000, $\eta_{p}^{2}$ = 0.82], but the main effect of load [*F*(1,17) = 0.24, *p* = 0.629, $\eta_{p}^{2}$ = 0.01] and the interactions were not significant [*F*(2,34) = 2.23, *p* = 0.123, $\eta_{p}^{2}$ = 0.12]. *Post hoc* comparisons (Bonferroni-corrected) for delay showed that when the delay in the dot’s response was longer, participants had greater difficulty achieving the goal (*M* = 1.51, 1.09, and 0.52 in the 100, 400, and 700-ms delay conditions, respectively; differences between all the conditions were significant, *p*s = 0.000). It is possible that cognitive load did not impair participants’ performance because the experimental task was not very difficult.

### Multivariate Analyses

**FIGURE 5:**
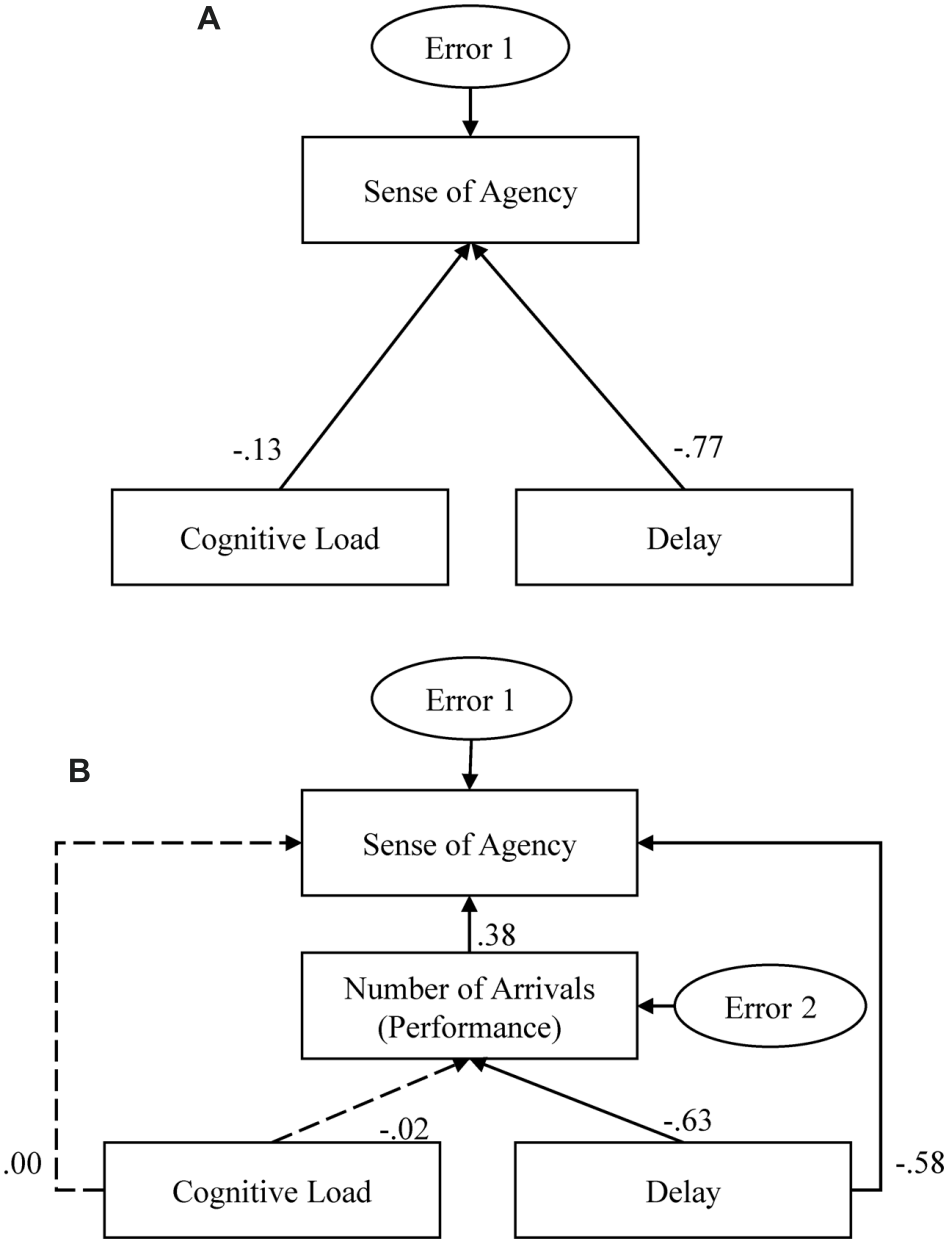
**Structural equation models and standardized regression weights for the **(A)** no-cued-goal and **(B)** cued-goal-directed conditions.** The paths with significant regression weights (predictions differed from zero at the 0.05 level, two-tailed) are represented by solid lines and the non-significant paths are represented by broken lines.

In order to examine the role of divided attention on the participant’s sense of agency over the moving dot and determine the extent of its influence, we conducted multivariate analyses for the two goal conditions. In the path model of the no-cued-goal condition (**Figure 5A**), level of cognitive load (low load = 0, high load = 1) and delay in response were included as independent variables, while agency ratings were included as a dependent variable. The dependent variable was directly influenced by the two independent variables, respectively. For the cued-goal-directed condition model (**Figure 5B**), the number of arrivals was included as an index of experimental task performance in addition to the three variables in the no-cued-goal condition model. Sense of agency was influenced by the independent (cognitive load and delay), and dependent variable (number of arrivals). Multivariate analyses therefore allowed us to estimate whether cognitive load and delay significantly influenced sense of agency directly or through other factors (e.g., task performance), and compare the extent to which variables influenced each other (e.g., the extent to which load and delay influenced sense of agency) between different goal conditions.

We performed structural equation modeling using IBM SPSS Amos 22. The fit parameters are shown in **Table 1**, and **Figure 5** includes the standardized coefficients for all paths (significant paths are represented by solid lines and non-significant paths are represented by broken lines). The two models demonstrate very good fit (**Table 1**). For the no-cued-goal condition model, both cognitive load and delay significantly influenced sense of agency (*p* = 0.034 and *p* < 0.001, respectively), and delay exerted a stronger influence. For the cued-goal-directed condition model, delay significantly and directly influenced sense of agency (*p* < 0.001), but cognitive load did not (*p* = 0.979). Furthermore, delay indirectly influenced sense of agency through task performance (path from delay to performance: *p* < 0.001; path from performance to sense of agency: *p* < 0.001).

**Table 1:** Fit parameters for the structural equation models for the no-cued-goal and cued-goal-directed conditions.

Model	χ^2^/df	GFI	AGFI	NFI	CFI	RMSEA
No-cued-goal	0.000	1.000	1.000	1.000	1.000	0.000
Cued-goal-directed	0.000	1.000	1.000	1.000	1.000	0.000
Good fit parameters	<2.0	≥0.90	≥0.90	≥0.90	≥0.90	≤0.08


*GFI, Goodness of Fit Index; AGFI, Adjusted Goodness of Fit Index; NFI, Normed Fit Index; CFI, Comparative Fit Index; RMSEA, Root Mean Square Error of Approximation.*

## Discussion

In the present study, we examined the influence of cognitive load on sense of agency with and without a cued goal. In the absence of a cued goal, actions were driven by internal goals; the action-effect comparison may have influenced the judgment of agency; our results showed that divided attention impaired sense of agency in this condition. In contrast, when actions were driven by an externally cued goal, the cued goal and feedback on goal attainment were salient cues for sense of agency; thus, processes relying on these cues were unaffected by attentional resources.

When there was no cued goal, participants probably acted upon internal goals. In this case, the judgment of agency probably relied on comparisons between one’s actions and their corresponding effects. According to the comparator model, comparisons rely on predictions based on efference copies of motor signals. When cognitive load was high in the no-cued-goal condition, both the predictions of effects and comparisons between prediction and actual sensory effects would have been more difficult, resulting in a poorer sense of agency. However, it is difficult to determine which aspect of the comparison processes was impaired by divided attention. Additionally, since we used a self-report paradigm it is not possible to determine whether cognitive load disrupted sense of agency itself, agency ratings, or comparison processes. The impact of cognitive load should be investigated further using different measures of sense of agency. Additionally, in the cued-goal-directed condition, delay did not interact with load. Thus, the insufficiency of attentional resources appears to contribute to the difficulty in action-effect comparisons in a particular manner, rather than only increasing the possibility of failure in matching. Hon et al. (2013) have also found decrements in participants’ judgment of agency for tasks with high cognitive load, however, this phenomenon was not observed when the delay of the effect was longer than 100 ms. This may have occurred because Hon et al. (2013) used a simpler measure for judging sense of agency; participants compared a single action with an effect, receiving feedback only once. In the present experimental task, participants had to control the moving dot continuously for a specific duration, give multiple commands, and compare their commands with continuous feedback. Therefore, the current comparison task was more difficult, as it required more cognitive resources compared to Hon et al.’s (2013) study, allowing us to detect the influence of divided attention. In addition, Haggard and Cole (2007) found that the intentional binding effect—the compression of the temporal percept between actions and effects—is stronger when attention is divided between events than that when attention is focus on one event. The intentional binding effect is considered to reflect implicit aspects of sense of agency (Moore and Obhi, 2012). Therefore, based on Haggard and Cole’s (2007) results, we can infer that divided attention improves implicit aspects of sense of agency. However, the link between the intentional binding effect and sense of agency remains controversial (Ebert and Wegner, 2010; Wen et al., 2015a), and the influences of divided attention on sense of agency and intentional binding effect could result from different processes. Nonetheless, the link between sense of agency and intentional binding and the properties of their underlying processes involving attention need to be explored in greater detail.

Further, we observed that when participants controlled the dot with a cued goal and received feedback on goal attainment, the availability of cognitive resources had no influence on the judgment of agency. When actions were driven by an external cued goal and feedback on goal attainment was provided, the comparison between the cued-goal and feedback of goal attainment probably was a salient cue for sense of agency, and the underlying processes that used this cue probably did not demand attention. That is, when feedback on goal attainment was available, it served as a constant external cue for the judgment of agency, while the internal cue—action-effect comparison—was impaired by cognitive load and had a weaker influence. The multivariate analyses results (**Figure 5**) also showed that the direct influence of delay on sense of agency in the cued-goal-directed condition was weaker than in the no-cued-goal condition (-0.58 vs. -0.77), and delay indirectly influenced the sense of agency via task performance. Therefore, the differing influences of divided attention on sense of agency in the two goal conditions may be attributable to the changing dominance of underlying processes in the judgment of agency. In addition, a limitation of the present study is that although the action-effect comparison had a weaker influence on the judgment of agency in the cued-goal-directed condition compared to the no-cued-goal condition, the action-effect comparison in the judgment of agency was not completely controlled in the cued-goal-directed condition. Therefore, in order to conclusively state that performance-based inference is automatic, a condition without a visible moving target and where only feedback on performance is provided should be included in future studies.

Alternatively, the presence of a goal may have increased attention to the experimental task (dot-controlling task), preventing the effect of cognitive load. However, we believe that this was not the case, because attentional capacity is considered to be limited (Baddeley, 2003). If participants shifted their attention from the digit memory task to the experimental task in the cued-goal-directed condition, their performance in the digit memory task should have decreased. However, performance in the digit task did not differ between the performance-based and no-cued-goal conditions (**Figure 3**), suggesting that the participants divided their attention in the same manner in the different goal conditions, as instructed by the experimenter.

In addition, we found that participants felt that they had less control when the goal was cued compared to when the goal was absent. This suggests that developing a sense of agency based on performance-based inferences compared to action-effect comparisons may be more difficult, although the former requires fewer cognitive resources. These results were consistent with a recent study that examined the influence of the goal on sense of control (Wen et al., 2015b). When a goal is present, sense of control is influenced by the comparison of the expectation of goal achievement and actual performance; consequently, the existence of a goal impairs sense of control when feedback is inconsistent with one’s expectations (Wen et al., 2015b). The influence of performance-based inference should be examined further by excluding action-effect comparison in the judgment of agency.

In summary, using a dual-task continuous control paradigm, we found that action-effect comparison underlying sense of agency is a controlled process, while the performance-based inference is an automatic process. The present study provides important insights that clarify the mechanism of sense of agency.

## Author Contributions

WW, AY, and HA designed this work. WW performed the experiment and analyses. WW, AY, and HA wrote the manuscript.

## Conflict of Interest Statement

The authors declare that the research was conducted in the absence of any commercial or financial relationships that could be construed as a potential conflict of interest.
